# Effectiveness of foot care guidelines on preventive knowledge and self-care behaviors among patients with diabetic neuropathy: a quasi-experimental study

**DOI:** 10.3389/fmed.2026.1818326

**Published:** 2026-06-16

**Authors:** Fatma Abdou Eltaib, Ibrahim Naif Alenezi, Ajitha Thankarajan Rajennal, Deepa Jothirajan, Lobna Mohamed Mohamed Abu Negm

**Affiliations:** 1 Medical Surgical Nursing Department, Faculty of Nursing, Ain Shams University, Cairo, Egypt; 2 Medical Surgical Nursing Department, Faculty of Nursing, Northern Border University, Arar, Saudi Arabia; 3 Public Health Nursing Department, College of Nursing, Northern Border University, Arar, Saudi Arabia; 4 Maternal and Child Health Nursing Department, Faculty of Nursing, Northern Border University, Arar, Saudi Arabia; 5 Emergency and Intensive Care Nursing Department, Faculty of Nursing, Northern Border University, Arar, Saudi Arabia

**Keywords:** foot care, health knowledge, neuropathies, patient education, practice, self-care behavior

## Abstract

**Background:**

Diabetic neuropathy, a debilitating complication of diabetes mellitus caused by prolonged hyperglycemia, is a significant chronic health problem in Saudi Arabia. This study aimed to evaluate the effectiveness of structured foot care guidelines in improving preventive knowledge and self-care practices among patients with diabetic neuropathy.

**Methods:**

This quasi-experimental, single-group pre- and post-intervention study was conducted from October 2023 to September 2024 at the Specialized Diabetes Center of Prince Abdel Aziz Bin Messad Hospital in Arar, Northern Border Region, Kingdom of Saudi Arabia. The study involved 40 adults with peripheral neuropathy selected through purposive sampling. The intervention included implementing the International Working Group on the Diabetic Foot (IWGDF) guidelines for diabetic patients with neuropathy, and data were collected using questionnaires to assess sociodemographic characteristics, adherence to foot care practices, and the presence of distal symmetrical peripheral neuropathy.

**Results:**

Statistical analysis using SPSS version 23 with descriptive and inferential statistical techniques showed a significant improvement (*p* < 0.02) in patients’ preventive foot care knowledge and self-care behaviors, along with improvements in neuropathy symptom reporting. The mean age of the participants was 65.35 ± 11.84 years; 65% were female patients and 35% were male patients.

**Conclusion:**

Structured foot care guidelines had a significant positive impact on patients’ knowledge, foot care behaviors, and reported neuropathy symptoms. These findings underscore the importance of ongoing educational and training programs to enhance foot care practices and increase awareness of the importance of reporting neuropathy-related complications.

## Introduction

Diabetes mellitus is a chronic metabolic disease characterized by hyperglycemia resulting from defective insulin secretion, insulin action, or both, leading to long-term damage to organs and tissues (1). The International Diabetes Federation (IDF) estimates that 588.7 million people are living with diabetes worldwide in 2024. In the absence of effective preventive strategies, this number is projected to reach 852 million by 2050 (2). Diabetes has become a major global health problem due to its increasing prevalence and long-term complications, which significantly affect the quality of life and place a substantial burden on healthcare systems.

According to the World Health Organization, the Kingdom of Saudi Arabia has the second-highest prevalence rate of diabetes in the world and is ranked second in the Middle East. Currently, nearly 7 million people have been diagnosed with diabetes, and approximately 3 million are in the pre-diabetic stage (3). Saudi Arabia is one of the 21 countries in the North Africa and Middle East region with an adult diabetes prevalence of 21.4% in 2024, according to the International Diabetes Federation (IDF) (4). The increasing burden of diabetes in Saudi Arabia has contributed to a rise in the incidence of diabetes-related complications, such as diabetic peripheral neuropathy and diabetic foot disorders.

Diabetic peripheral neuropathy (DPN) is a debilitating chronic microvascular complication of diabetes. A systematic review and meta-analysis conducted in Saudi Arabia concluded that the overall prevalence of DPN was 39%, emphasizing the significant health burden it imposes on patients with diabetes (5). A cross-sectional study conducted in Saudi Arabia on DPN reported a prevalence of 30.1% among patients with type 2 diabetes mellitus and 25.9% among those with type 1 diabetes. The study found significant associations between DPN and factors such as advanced age, duration of diabetes, uncontrolled HbA1c levels, and family history (6). Other risk factors for DPN include abdominal obesity, elevated fasting blood glucose levels, high creatinine levels, and increased white blood cell counts, whereas the appropriate use of oral hypoglycemic medication has been associated with a lower risk (7).

Diabetic neuropathy (DN) is one of the most common neuropathic complications in patients with diabetes, affecting more than 50% of patients during their lifetime (8). Common clinical findings include pain, burning, tingling, and numbness of the feet and toes. Gradually, these symptoms extend to the legs and hands as the disease progresses (9). Peripheral diabetic neuropathy can cause serious complications, such as foot ulceration, infection, gangrene, and lower limb amputation (10, 11). Patients with diabetes are at an increased risk of ischemia, gangrene, multi-organ failure, peripheral vascular disease, sensory loss, and death associated with diabetic neuropathy (12).

Diabetic foot ulcers are one of the most serious yet preventable complications of diabetes and remain a major cause of lower-extremity amputation (9, 13). Internationally, the prevalence of diabetic foot complications ranges from 4.6 to 12%. The risk factors include sex, age, duration of diabetes, poor glycemic control as indicated by increased HbA1c levels, and smoking (11). The prevalence of diabetic foot complications among patients with diabetes in Saudi Arabia has been reported to be 3.3% (4), reflecting the growing clinical burden in the country.

Nurses play a key role in preventing diabetic foot complications in at-risk patients. This may be achieved through patient health education, regular foot examinations, early risk screening, and the development of self-care practices (14). Patient education is an effective method for preventing diabetic foot ulcers and improving patient outcomes. The International Working Group on the Diabetic Foot (IWGDF) has developed five key recommendations for the prevention of diabetic foot: early identification of at-risk feet, regular foot examination, patient and family education, appropriate use of therapeutic footwear, and management of modifiable risk factors for foot ulcer (15).

Despite the availability of the International Working Group on the Diabetic Foot (IWGDF) guidelines, a gap remains in the effective implementation of structured diabetic foot care education among patients with diabetic peripheral neuropathy, especially in primary healthcare centers in Saudi Arabia (16). Previous studies conducted in Saudi Arabia among patients with diabetes have mainly assessed knowledge of foot care and foot care practices, reporting insufficient knowledge and poor self-care practices (17). However, there is scant evidence on the effectiveness of structured guideline-based interventions in improving knowledge and foot care practices among patients with diabetic peripheral neuropathy (18).

Moreover, there have been limited research studies specifically reported among patients residing in the Northern Border Region of Saudi Arabia. Variations in accessible healthcare services, patient education, and primary preventive strategies influence diabetes-related outcomes. Hence, this study is novel in its assessment of the effectiveness of structured foot care guidelines in patients with diabetic peripheral neuropathy. Therefore, this study aimed to evaluate the effectiveness of structured foot care guidelines in enhancing preventive knowledge and self-care behaviors among patients with diabetic peripheral neuropathy in the Northern Border region of Saudi Arabia. We hypothesized that implementing foot care guidelines would significantly improve patients’ knowledge and foot care behaviors compared to pre-intervention levels.

## Materials and methods

### Study design and setting

This study utilized a quasi-experimental, single-group pre-test/post-test design to evaluate the impact of foot care guidelines for diabetic patients with neuropathy. This design is appropriate for assessing outcomes in clinical settings where randomization may not be feasible (19). The research was conducted at the specialized Diabetes Center of Prince Abdel Aziz Bin Messad Hospital in Arar, Northern Border Region, Kingdom of Saudi Arabia, which serves a large number of patients with diabetic complications.

### Participants and sampling

A purposive sampling technique was employed to recruit 40 adults with diabetes. Recruitment and data collection spanned from October 2023 to September 2024.

### Eligibility criteria

Inclusion Criteria: Adults (≥18 years) of both sexes with a confirmed diagnosis of type 1 or type 2 diabetes and clinically diagnosed peripheral neuropathy.

Exclusion Criteria: Patients with active diabetic foot ulcers, a history of lower limb amputation, active foot infections, or those who had participated in a formal foot care education program in the past 6 months.

### Sample size calculation

The sample size was determined using the Epical 2000 software. Based on a 5% level of significance (*α* = 0.05), power of 90% (*β* = 0.10), and an expected medium effect size in knowledge and practice scores, a minimum sample of 40 participants was required to ensure statistical robustness.

### Data collection instruments

Data were collected using three standardized instruments and a researcher-developed questionnaire. All tools were administered through structured face-to-face interviews to ensure data quality and completeness.

1 Sociodemographic and Clinical Profile.

A structured questionnaire was used to collect the following data:

- Demographics: Age, sex, marital status, educational level, and occupation.
- Clinical history: Type of diabetes, duration of illness, current pharmacological management (oral agents vs. insulin), and presence of comorbidities (e.g., hypertension and dyslipidemia).
- Health status indicators: Adherence to a diabetic diet, frequency of annual foot screenings, body mass index (BMI), and most recent glycated hemoglobin (HbA1c) levels.

2 Diabetic Foot Care Knowledge Questionnaire.

This 20-item tool assessed knowledge across five domains: daily foot care, inspection techniques, footwear selection, injury management, and lifestyle modifications (smoking cessation and medication adherence) (20, 21).

- Scoring: Each correct response received 1 point and incorrect or “do not know” responses received 0 points.
- Interpretation: The total scores ranged from 0 to 20. Scores ≥12 (60%) were categorized as “satisfactory knowledge,” while scores <12 were “unsatisfactory.”
- Reliability: The tool underwent pilot testing, yielding a Cronbach’s alpha of 0.82, indicating high internal consistency.

1. 3 Nottingham Assessment of Functional Footcare (NAFF).

The NAFF is a 29-item standardized instrument that measures the frequency of protective foot care behaviors (22).

- Scoring: Items are scored on a 4-point Likert scale (0 = never, 1 = rarely, 2 = sometimes, 3 = often). The total score ranged from 0 to 87.
- Interpretation: Higher scores indicate better adherence to foot care practices. A threshold of ≤50 is used to identify patients requiring intensive behavioral intervention (23).
- Psychometric properties: The original version reported a Cronbach’s alpha of 0.53 (22). Although lower than typical thresholds, this is considered acceptable for the NAFF because it measures a heterogeneous set of independent behaviors (e.g., shoe checking vs. toe drying) rather than a single psychological trait. It demonstrates excellent test–retest reliability (*r* = 0.89).

4 Michigan Neuropathy Screening Instrument (MNSI).

The MNSI is a gold standard tool for identifying distal symmetrical peripheral neuropathy (24).

- Part A (Patient History): A 15-item self-report questionnaire. A score of 1 is given for “Yes” responses on items 1–4, 5–6, 8–10, 11–12, 14–15, and for “No” responses on items 7 and 13. A score of ≥7 is considered abnormal.
- Part B (Physical Examination): A clinical assessment of the feet, including

◦ Visual inspection (appearance of feet): Deformities, dry skin, calluses, or fissures (1 point per foot).
◦ Ulceration: Presence of ulcers (1 point per foot).
◦ Ankle reflexes: Scored as 0 (present), 0.5 (present with reinforcement), or 1 (absent).
◦ Vibration sensation: Tested at the great toe using a 128-Hz tuning fork. The score was 0 (present for >10s), 0.5 (reduced, 10s), or 1 (absent).

- Interpretation: The maximum examination score was 8, and a score >2.5 was highly suggestive of neuropathy. The MNSI has shown high sensitivity (80%) and specificity (95%) in clinical trials (25, 26).

### Research intervention: foot care guidelines

The intervention consisted of a structured educational program titled “Foot Care Guidelines for Neuropathy Patients,” developed based on the International Working Group on the Diabetic Foot (IWGDF) guidelines (15).

#### Delivery protocol

- Duration: Four consecutive weeks.
- Frequency: every session takes 45-min per week.
- Format: Small group sessions (6–8 participants) to facilitate interaction.
- Teaching methods:

◦ Visual aids: Interactive PowerPoint presentations and high-resolution clinical images.
◦ Demonstration: Hands-on training for foot inspection using a mirror and proper nail trimming.
◦ Printed materials: A detailed illustrated brochure in Arabic was provided to each participant for home reference.
◦ Feedback: Q&A sessions at the end of each meeting to address individual concerns.

#### Content modules

- Week 1: Pathophysiology of diabetes and neuropathy and identification of sensory loss.
- Week 2: Daily foot hygiene, including washing, drying (especially between the toes), and moisturizing techniques.
- Week 3: Footwear science; criteria for choosing therapeutic shoes and the “shake-out” technique.
- Week 4: Risk management, when to contact a healthcare provider, and emergency foot care.

#### Intervention and data collection method

Data were systematically collected from the end of October 2023 to the end of September 2024 at the Diabetic Center in Arar City, Northern Border Region, Kingdom of Saudi Arabia. Participants were selected based on predefined inclusion criteria to ensure homogeneity and relevance to the study’s objectives. The data collection process was meticulously structured into three distinct phases: baseline assessment (pre-intervention), post-intervention assessment, and comprehensive follow-up assessment.

#### Baseline assessment (pre-intervention)

A baseline assessment was conducted during the initial week for each participant group. This involved a structured interview administered by the researcher, which included questionnaires covering sociodemographic data and participants’ existing knowledge of foot care. The Michigan Neuropathy Screening Instrument (MNSI) was used to objectively assess the presence and severity of distal symmetrical peripheral neuropathy. Adherence to the recommended foot care practices was evaluated using the Nottingham Assessment of Functional Footcare (NAFF) tool. All baseline data collection was standardized across all participants to minimize interviewer bias and ensure comparability of initial characteristics (Table 1).

**Table 1:** Pre-intervention phase.

Week	Session objective	Main content	Delivery method	Duration	Participants	Setting
Week 1	Assessment session	Fostering intimacy in relationships by being friendly and attentive during the initial meeting. Through the meeting, sociodemographic data were collected Michigan and Nottingham tools were applied to detect the level of neuropathy	Face-to-face interview to assess the level of neuropathy through standardized instruments	15 min/ patient in assessment	(with 6–8 patients)	Diabetic center

#### Intervention phase

Following the baseline assessment, participants engaged in the 4-week structured educational program, “Foot Care Guidelines for Neuropathy Patients,” as detailed in the Intervention Session Schedule. During this phase, the researchers provided verbal encouragement to foster consistent engagement in diabetes management and foot self-care behaviors. While no formal data were collected during the intervention sessions, participant engagement and understanding were assessed informally through Q&A sessions and hands-on demonstrations (Table 2).

**Table 2:** Intervention phase.

Week	Session objective	Main content	Delivery method	Justification for delivery methods	Duration	Participants	Setting
Week 2	Understand the basics of diabetic neuropathy and identify sensory loss.	Pathophysiology of diabetes and neuropathy: identifying sensory loss.	Interactive PowerPoint presentations, high-resolution clinical images, and Q&A sessions.	Facilitating ongoing communication and support between sessions, thereby enhancing adherence to the program	45 min	6–8 patients	Diabetic center
Week 3	Learn daily foot hygiene practices to maintain foot health and understand the importance of therapeutic footwear and selection criteria	Daily foot hygiene: washing, drying (especially between toes), and moisturizing techniques. And footwear science: criteria for choosing therapeutic shoes and the “shake-out” technique.	Interactive PowerPoint presentations, high-resolution clinical images, hands-on training, and Q&A sessions.		45 min	6–8 patients	Diabetic center
Week 4	Recognize risk signs and when to contact a healthcare provider.	Risk management: when to contact a healthcare provider and emergency foot care.	Interactive PowerPoint presentations, high-resolution clinical images, and Q&A sessions.		45 min	6–8 patients	Diabetic center
Week 5 to Week 24	Follow-up	Motivation for self-management in participants’ daily lives through encouraging participants to continuously plan to achieve all foot self-care behaviors.	The participants could ask the questions via private chat if they needed to protect the privacy and confidentiality of the research participants.	The ability to provide personalized reminders and additional educational materials through these platforms.	Any time	Individualized	Via private chat

#### Post-intervention assessments

Immediately following the completion of the 4-week intervention (at Week 5), participants were given the opportunity to address any remaining questions, reinforcing the educational content. Post-intervention assessment was performed 20 weeks after the intervention, totaling 25 weeks from the study’s commencement. Post-intervention assessments mirrored the baseline assessment, utilizing the same standardized tools (knowledge questionnaires, MNSI, and NAFF) to evaluate changes in knowledge, adherence to foot care practices, and the status of distal symmetrical peripheral neuropathy. This consistent application of assessment tools across all phases ensured robust data for evaluating the short-term and sustained effects of the intervention. Measures to minimize recall bias included clear instructions and standardized administration of the questionnaires at each assessment point (Table 3).

**Table 3:** Post-intervention phase.

Week	Session objective	Main content	Delivery method	Justification for delivery methods	Duration	Participants	Setting
Week 25	Assessment session	Fostering intimacy in relationships by being friendly and attentive during the initial meeting. Assess the level of patients’ knowledge Michigan and Nottingham tools were applied to detect the level of neuropathy	Face-to-face interview to assess the level of neuropathy through standardized instruments	To assess facilitators’ adherence to the protocol and provide feedback for continuous improvement.	15 min/ patient in assessment	(with 6–8 patients)	Diabetic center

### Statistical analysis

All data were meticulously entered and subsequently analyzed utilizing IBM SPSS Statistics, Version 23. Descriptive statistics, including frequencies and percentages, were employed to characterize the study population and to present the primary findings in both tabular and graphical formats. For the analysis of paired categorical data within contingency tables, the McNemar test was applied to assess the significance of changes in proportions. The Pearson Product–Moment Correlation Coefficient (r) was computed to quantify the strength and direction of linear relationships between continuous quantitative variables. Furthermore, paired-samples t-tests were utilized to compare mean differences in self-care behaviors before and after the intervention, particularly in relation to sociodemographic and medical characteristics. Statistical significance was prospectively defined as a two-sided *p*-value <0.05. *p*-values exceeding this threshold (*p* > 0.05) were considered non-significant, while those falling below (*p* < 0.05) were deemed statistically significant.

### Ethical considerations

This study was approved by the Bioethics Committee of Northern Border University (HAP-09-A-043), No. (75/44/H). Before participating in this study, the research objectives, data collection procedures, the benefits and impact of the study, and the right to participate or withdraw without any effect on care were explained. All participants willingly signed an informed consent form. All data were kept confidential and presented in terms of the overall outcomes.

## Results

Table 4 illustrates the sociodemographic characteristics of patients; approximately two-thirds (62%) were in the age range of 48–67 years and 65% were female patients. In addition, 92.5% were married, 87.5% had primary education, and 72.5% were unemployed.

**Table 4:** Frequency and percentage distribution of patients with diabetic neuropathy demographic features (*n* = 40).

Variables	Studied patients	
	No.	%
Age (Years)		
28–47	9	22.5
48–67	25	62.5
Over 67	6	15.0
Mean ±SD	65.35 ± 11.84	
Sex		
Male	14	35.0
Female	26	65.0
Marital status		
Married	37	92.5
Widow	1	2.5
Separated	2	5.0
Education		
Primary education	35	87.5
Secondary education	3	7.5
University education	2	5.0
Occupation		
Yes	11	27.5
No	29	72.5

Table 5 demonstrates the past and present history of patients with diabetic neuropathy. The majority of patients (90%) had type II DM, and nearly half of them (40%) had a history of diabetes mellitus for 6–10 years. More than half (52.5%) were on oral hypoglycemic drugs only, had no other chronic diseases, and had a normal body mass index. A total of 55% of the patients stated that they had good control over their blood glucose level, while the mean ±SD of HbA1c was 8.22 ± 1.23. The majority of patients (92.5%) underwent annual check-ups, and 87.5% followed a special diet.

**Table 5:** Frequency and percentage distribution of diabetic neuropathy patients’ medical history (n = 40).

Variables	Studied patients	
	No.	%
Diabetes mellitus category		
Type 1	4	10
Type 2	36	90
Diabetes mellitus duration		
less 5 years	3	7.5
6–10 years	16	40
11–15 years	14	35
more than 15 years	7	17.5
Diabetes mellitus: Current treatment		
Oral hypoglycaemic drugs only	21	52.5
Insulin only	8	20.0
Both insulin and oral hypoglycemic drugs	11	27.5
Presence of another chronic disease		
No	21	52.5
Yes	19	47.5
Follow a special diet		
Yes	35	87.5
No	5	12.5
Perform an annual check-up		
Yes	37	92.5
No	3	7.5
Blood Glucose Level		
Poor control	18	45
Good control	22	55
BMI		
Normal 18.5 ≤ 25	21	52.5
Overweight 25 ≤ 30	18	45
Obese < 30	1	2.5
Mean ±SD	20.8875 ± 12.38873	
HBa1c		
Good control (>5.7–6.4)	17	42.5
Poor control <)6.5)	23	57.5
Mean ±SD	8.2200 ± 1.22625	

Table 6 changes in foot care knowledge before and after implementing foot care guidelines. In relation to patient knowledge, the majority of the patients (57.5, 62.5, 65, 77.5, and 77.5%) had unsatisfactory knowledge about when to inspect the foot, the risk factors for developing foot ulcers, daily foot care, suitable footwear, and warning signs and symptoms of foot ulcers, respectively, with a total (60%) having unsatisfactory knowledge. After the implementation of the foot care guidelines, most of the patients (97.5, 87.5, 85, and 75%) had satisfactory knowledge about the same items, except that more than half of them (55%) had satisfactory knowledge about warning signs and symptoms of foot ulcers, with 85% demonstrating satisfactory total knowledge.

**Table 6:** Difference between patients’ knowledge of foot care pre- and post-intervention of foot care guidelines (n = 40).

Knowledge	Pre (*n* = 40)		Post (*n* = 40)		McNemar test *p*-value
	Unsatisfactory	Satisfactory	Unsatisfactory	Satisfactory	
Patient knowledge about when to inspect the foot	23 (57.5%)	17 (42.5%)	1 (2.5%)	39 (97.5%)	<0.001
Patient knowledge about the risk factors of developing foot ulcers	25 (62.5%)	15 (37.5%)	5 (12.5%)	35 (87.5%)	<0.001
Patient knowledge about daily foot care	26 (65%)	14 (35%)	6 (15%)	34 (85%)	<0.001
Patient knowledge about suitable footwears	31 (77.5%)	9 (22.5%)	10 (25%)	30 (75%)	<0.001
Patient knowledge about warning signs and symptoms of foot ulcers	31 (77.5%)	9 (22.5%)	18 (45%)	22 (55%)	0.002
Total knowledge	24 (60%)	16 (40%)	6 (15%)	34 (85%)	<0.001

Table 7 illustrates patients’ functional foot care behaviors pre- and post-educational intervention regarding foot care. The highest percentage of patients (67.5%) had poor compliance with the recommended foot care behaviors pre-intervention. While 92.5% of the participants had good compliance with recommended foot care behaviors post-intervention, there was a statistically significant difference between pre- and post-intervention (*p*<0.001).

**Table 7:** Comparison between functional foot care behaviors of the studied patients pre- and post-intervention of foot care guidelines (*n* = 40).

Items	Pre (*n* = 40)		Post (*n* = 40)		*McNemar test*
	Poor self-care	Good self-care	Poor self-care	Good self-care	*p*-value
The patient examines their feet?	15 (37.5%)	25 (62.5%)	5 (12.5%)	35 (87.5%)	0.031
The patient checks their shoes when they put them on and take them off	24 (60%)	16 (40%)	11(27.5%)	29 (72.5%)	0.002
The patient washes their feet and dries them well, especially between toes	19 (47.5%)	21 (52.5%)	9 (22.5%)	31(77.5%)	0.052
The patient use moisturizing cream on their feet?	23 (57.5%)	17 (42.5%)	12 (30%)	28 (70%)	0.019
The patient put moisturizing cream between their toes?	27 (67.5%)	13 (32.5%)	16 (40%)	24 (60%)	0.035
The patient has their toenails cut?	17 (42.5%)	23 (57.5%)	7 (17.5%)	33 (82.5%)	0.041
The patient wears inappropriate shoes such as sandals, slippers, and lace-up shoes	26 (65%)	14 (35%)	15 (37.5%)	25 (62.5%)	0.007
The patient breaks in new shoes gradually	26 (65%)	14 (35%)	14 (35%)	26 (65%)	0.008
The patient wears artificial fiber (e.g., nylon) socks?	19 (47.5%)	21 (52.5%)	8 (20%)	32 (80%)	0.001
The patient wears seamless socks and changes them often	31 (77.5%)	9 (22.5%)	10 (25%)	30 (75%)	<0.001
The patient wears shoes without socks	20 (50%)	20 (50%)	8 (20%)	32 (80%)	0.008
The patient walks barefoot in the house and out of the house	17 (42.5%)	23 (57.5%)	7 (17.5%)	33 (82.5%)	0.021
The patient uses tools for heating the foot, such as hot water bottle, near the fire, and radiator.	22 (55%)	18 (45%)	7 (17.5%)	33 (82.5%)	<0.001
The patient uses a bath thermometer	22 (55%	18 (45%)	13 (32.5%)	27 (67.5%)	0.078
The patient uses corn remedies/corn plasters/paints when they get a corn	17 (42.5%)	23 (57.5%)	14 (35%)	26 (65%)	0.678
The patient put a dry dressing on a blister, graze, cut, or burn	38 (95%)	2 (5%)	19 (47.5%)	21 (52.5%	<0.001
Total self-care behaviors	27 (67.5%)	13 (32.5%)	3 (7.5%)	37 (92.5%)	<0.001

**Figure 1:**
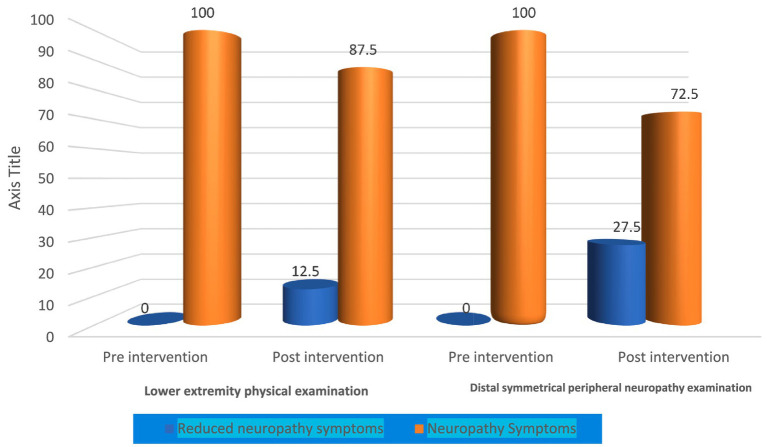
Comparison of peripheral neuropathy among patients with diabetic neuropathy pre- and post-intervention (*n* = 40).

Figure 1 illustrates the prevalence of distal symmetrical peripheral neuropathy (DSPN) among patients before and after the intervention. Before the intervention, all patients (100%) presented with discernible symptoms of neuropathy. Following the intervention, the detection rate of neuropathy symptoms significantly decreased to 87.5%, demonstrating a statistically significant difference (*p* < 0.02). Furthermore, a notable improvement was observed in the reporting of neuropathy symptoms during lower extremity examination, with prevalence decreasing from 100% pre-intervention to 72.5% post-intervention. This reduction was associated with a highly statistically significant difference (*p* < 0.0001).

Table 8 shows the correlation between the knowledge level and functional foot care behaviors in the studied patients pre- and post-intervention; less than one-quarter of patients (12.5%) with a satisfactory knowledge level of foot care had good foot self-care behaviors pre-intervention, which improved to 77.5% of patients with satisfactory knowledge having good foot self-care behaviors post-intervention, with a highly statistically significant difference between pre- and post-intervention (*p* = 0.023).

**Table 8:** Correlation between the knowledge level and functional foot care behaviors in studied patients pre- and post-intervention of foot care guidelines.

Knowledge level	Pre-intervention (40)		Post-intervention (40)		*Pearson’s r*	*p*-value
	Poor foot self-care behaviors	Good foot self-care behaviors	Poor foot self-care behaviors	Good foot self-care behaviors		
Satisfactory level	11 (27.5%)	5 (12.5%)	3 (7.5%)	31(77.5%)	0.253	0.023
Unsatisfactory level	16 (40%)	8 (20%)	0 (0%)	6 (15%)		

Table 9 shows the association between diabetic foot self-care behaviors and sociodemographic characteristics of patients with diabetic neuropathy. There was a highly significant difference between patients’ foot self-care behaviors and the 48–67 years age group, female patients, marital status, and primary education level.

**Table 9:** Relation between diabetic foot self-care behaviors and sociodemographic characteristics among patients with diabetic neuropathy pre- and post-intervention.

Variables	Pre-intervention	Post-intervention	T	*p*-value
	Self-care behaviors Mean +SD	Self-care behaviors Mean +SD		
Age (Years)				
28–47	50.11 ± 5.51	60.67 ± 5.7	3.99	0.001
48–67	46.68 ± 6.74	62.4 ± 6.55	8.36	0.000
Over 67	47.67 ± 4.63	59.67 ± 9.63	2.75	0.02
Sex				
Male	48.93 ± 5.89	62 ± 4.94	6.36	0.000
Female	46.88 ± 6.41	61.38 ± 7.69	7.38	0.000
Marital status				
Married	47.29 ± 6.16	61.43 + 7.02	9.2	0.00
Widow	43	63	-	-
Separated	55.5 + 0.71	64 + 2.83	4.12	0.054
Level of education				
Primary education	47.02 + 6.24	61.8 + 7.09	9.25	0.000
Secondary education	51.33 + 6.42	60.67 + 5.13	1.96	0.12
University education	52 + 4.24	59.5 + 4.95	1.62	0.24
Occupation				
Yes	49.18 + 7.49	63.09 + 5.03	5.11	0.000
No	47 + 5.72	61.03 + 7.35	8.11	0.000

Table 10 shows the relationship between diabetic foot self-care behaviors and patients’ medical characteristics before and after the educational intervention of foot care guidelines. There was a highly significant difference between patients’ foot self-care behaviors and type II diabetes mellitus, a diabetes duration of 6–10 years, use of oral hypoglycemic treatment only or insulin only, absence of other chronic diseases, adherence to a special diet, annual check-up attendance, and overweight status (BMI ≥ 25–≥30).

**Table 10:** Relation between diabetic foot self-care behaviors and studied patient medical data pre and post-intervention of foot care guidelines.

Variables	Pre-intervention	Post-intervention	T	*p*-value
	Self-care behaviors Mean +SD	Self-care behaviors Mean +SD		
Diabetes mellitus category				
Type 1	43 + 1.825	59 + 2.16	11.3116	0.000
Type 2	48.11 + 6.355	61.89 + 7.08	8.77	0.000
Diabetes mellitus duration				
less than 5 years	51 + 3.464	59.67 + 3.511	3.05	0.038
6–10 years	46.87 + 6.89	62.81 + 6.85	6.55	0.000
11–15 years	48.42 + 7.14	60.71 + 7.52	4.43	0.000
more than 15 years	46.14 + 2.85	61.42 + 6.94	5.55	0.000
Diabetes mellitus: current treatment				
Oral hypoglycemic drugs only	48.67 + 6.191	64 + 5.88	8.22	0.000
Insulin only	42.12 + 4.97	59.5 + 4.78	7.13	0.000
Both insulin and oral hypoglycemic drugs	49.54 + 5.222	58.54 + 8.29	3.05	0.0066
Presence of another chronic disease				
Yes	47.52 + 4.71	60.42 + 7.42	6.39	0.0000
No	47.67 + 7.48	62.67 + 6.15	7.1	0.000
Occupation				
Yes	49.18 + 7.49	63.09 + 5.03	5.11	0.000
No	47 + 5.72	61.03 + 7.35	8.11	0.000
Follow a special diet				
Yes	47.77 + 6.31	61.48 + 6.95	8.64	0.000
No	46.4 + 6.27	62.4 + 6.23	4.05	0.004
Perform an annual check-up				
Yes	48.11 + 6.15	61.46 + 6.82	8.84	0.000
No	41.33 + 3.78	63.33 + 7.51	4.53	0.011
Blood glucose level				
Good control	48.22 + 6.18	61.22 + 5.02	7.66	0.000
Poor control	46.83 + 6.4	62.05 + 8.62	6.01	0.000
BMI				
Normal 18.5 ≤ 25	46.1 + 6.45	58.8 + 6.18	6.35	0.000
Overweight 25 ≤ 30	49.15 + 5.93	64.05 + 6.29	7.74	0.000
Obese < 30	48	71	--	---

## Discussion

Diabetic neuropathy is among the most prevalent microvascular complications of diabetes and is a major contributor to diabetic foot ulcers (DFUs). Early detection and preventive interventions are essential, given the absence of disease-modifying therapies, as they can significantly reduce the risk of developing diabetic foot ulcers and improve overall patient outcomes. The present study assessed the effectiveness of evidence-based foot care guidelines for improving knowledge and self-care behaviors among patients with diabetic neuropathy.

The age of participants in the present study ranged between 48 and 67 years, which is in accordance with the fact that neuropathy is found at a higher prevalence with increasing age and duration of diabetes, as has been observed in previous studies (27). More than two-third of the participants were women, indicating that the prevalence of peripheral neuropathy in women with type II diabetes is higher (28). Most of them were married, which could be attributed to the participants’ age and is consistent with the literature (29). It was observed that the majority of patients had a primary level of education and were unemployed, which is different from other studies in which most patients had a higher level of education and employment (30). These sociodemographic factors may affect individual participation in educational sessions and the development of self-management skills.

Type II DM, with a 6–10-year duration, is treated with oral hypoglycemic agents with satisfactory glycemic control in most individuals (31). Differences in medication use patterns and the duration of diabetes likely depend on the nature of the diabetic population and the healthcare infrastructure.

The foot care knowledge of the patients improved significantly after applying foot care guidelines. In general, the patients were not knowledgeable about foot care before the intervention. However, after receiving the intervention, they achieved much improvement in knowledge, showing that through specific education, patients are empowered to take care of themselves, which has also been proven successful by other studies with marked knowledge improvement in similar conditions (32). The changes seen in self-care behavior also coincided with that of the knowledge, where the majority of patients improved their practice from poor to good foot care post-intervention. This also matched with previously researched information where evidence showed that educational intervention (using counseling, demonstration, and follow-up strategy) improved foot care practices and decreased DFU risk (33, 34). The health belief and self-efficacy models also support these results, as improved knowledge increased both the perception of susceptible risk and self-confidence to achieve appropriate foot care behavior.

However, reversal of neuropathy is unlikely to occur in the short term. The increase in reported symptoms and improvements in physical foot examination after the intervention were likely due to increased knowledge, prompt identification, and good compliance with preventive actions. This supports reports that routine foot screening and education can decrease both the self-reported prevalence and severity of neuropathic symptoms (35).

Knowledge scores correlated with behavior scores following the intervention. This indicates that knowledge is the key to behavioral change. This is in agreement with a study that found that increased knowledge of foot care is related to more frequent self-examination and preventive measures in patients with diabetes (36).

Foot self-care practices were statistically associated with age, sex, marital status, education, and employment status. Older patients (48–67 years) and women exhibited higher levels of foot self-care behaviors, which agrees with other studies (37, 38). The association between marital status and self-care behavior could be explained by the possible benefit of a partner’s participation in day-to-day self-care activities (39, 40). People with only primary education and unemployed individuals demonstrated higher levels of self-care behaviors, possibly due to having a less demanding life and more time for interventions (27). Nevertheless, these associations need to be considered carefully, as some of them have weaker backing from previous evidence.

A positive relationship was found between better foot care behaviors and factors such as diabetes duration, use of single treatment modalities (oral or insulin alone), and adherence to a diet for diabetes management. These findings suggest that longer disease duration and consistent medication routines may enhance patients’ self-management capacity through accumulated experience and continuous education (41–43).

The current research confirms our hypothesis that systematic and evidence-based guidelines on foot care effectively improve knowledge of prevention and self-care behaviors in patients with diabetes. Similar to previous systematic reviews and meta-analyses, education alone may improve knowledge and practices but may not result in sustained behavior changes without continuous education and encouragement (33, 44, 45). Hence, this indicates the necessity of embedding continuous education and behavior support in diabetic programs to prevent DFUs and the burden of neuropathy (46).

Overall, this intervention has been shown to be beneficial in enhancing both awareness and behavior among diabetic patients regarding the care of their feet. This may be attributed to increased health literacy, improved self-efficacy, and increased participation in proactive preventive care. Future studies are warranted to explore long-term adherence, specifically by incorporating theoretical models into the educational design and by measuring diabetic foot ulcer occurrence and quality of life at long-term follow-up.

### Study limitations

While this study provides valuable insights into the impact of foot care guidelines for patients with diabetic neuropathy, it is subject to several limitations that warrant consideration when interpreting the findings. These limitations primarily stem from the methodological design and contextual factors of the study.

First, adopting a single-group quasi-experimental design without a concurrent control group inherently limits the ability to establish definitive causal relationships between the intervention and the observed outcomes. While this design was chosen due to the practical constraints of a clinical setting where randomization might not be feasible (19), it precludes isolating the specific effects of the intervention from other confounding variables or natural progression. Consequently, any improvements in knowledge or practice cannot be unequivocally attributed solely to the educational program, as external factors or maturation effects may have contributed.

Second, the study was conducted within a single-center setting at the specialized Diabetes Center of Prince Abdel Aziz Bin Messad Hospital in Arar, Northern Border Region, Kingdom of Saudi Arabia. While this allowed for focused data collection and consistent intervention delivery, it significantly restricted the generalizability of the findings to other populations, healthcare systems, or cultural contexts. The specific patient demographics, healthcare resources, and prevalent practices within this single center may not be representative of the broader diabetic population, thus limiting the external validity of the results.

Third, reliance on self-reported measures to assess knowledge and foot care practices introduces a potential risk of reporting bias. Participants may have provided responses that they perceived as socially desirable or aligned with the study’s objectives rather than accurately reflecting their true knowledge or behaviors. Although measures were taken to minimize interviewer bias through the standardized administration of questionnaires, the subjective nature of self-reporting remains a concern, potentially leading to an overestimation of the intervention’s effectiveness.

Fourth, the Hawthorne effect may have influenced the participants’ responses and behaviors. The awareness of being observed and participating in a research study could have inadvertently led participants to alter their behavior or improve their adherence to foot care practices, irrespective of the educational intervention itself. This phenomenon could inflate the perceived impact of the guidelines, making it challenging to ascertain the true efficacy of the program in routine conditions.

Fifth, the short-term outcome assessment conducted 20 weeks post-intervention (25 weeks from study commencement) provides only a snapshot of the intervention’s effects. While this timeframe is adequate for evaluating immediate changes, it does not capture the long-term sustainability of improved knowledge or foot care practices. The durability of behavioral changes and their impact on clinical outcomes, such as the incidence of foot complications, remain unknown beyond this assessment period. Future research with extended follow-up periods is crucial to ascertain the lasting benefits of such interventions.

Finally, the use of a purposive sampling technique to recruit participants, while practical for targeting a specific patient group, introduced selection bias. This non-random approach means that the sample may not be fully representative of the broader population of patients with diabetes and neuropathy, further limiting the generalizability of the findings. The characteristics of the selected participants might differ systematically from those not included, potentially affecting the external validity of the study.

### Implication for nursing practice

The findings of this study offer practical guidance for nursing practice by informing nurses how to strengthen diabetic foot care behaviors through structured, guideline-based education and follow-up. First, nurses can play a central role in delivering patient-centered education tailored to patients’ knowledge levels and behavioral needs, emphasizing consistent foot inspection, appropriate hygiene practices, early recognition of foot complications, and adherence to preventive behaviors to reduce the risk of neuropathy and its progression.

Second, the results support integrating these educational interventions into routine primary care workflows. Embedding nurse-led foot care education within primary care settings can enhance continuity of care, promote timely referrals for patients at higher risk, and ensure that education is not a one-time event but an ongoing process reinforced at each visit. In this context, structured guideline-based interventions are essential to standardize care delivery, improve the quality and completeness of patient education, and reduce variability in the communication of foot care recommendations.

To ensure the applicability and transferability of these practice implications, further research using a larger probability sample across multiple provinces in Saudi Arabia is recommended. This will help confirm whether the identified educational and behavioral priorities are consistent across diverse patient populations and healthcare contexts, thereby supporting the broader implementation of nurse-led, structured foot care interventions at the national level.

## Conclusion

In this quasi-experimental, single-group, pre–post study, foot care guideline implementation for patients with diabetic neuropathy resulted in a statistically significant improvement in patients’ preventive foot care knowledge and behaviors. These findings underscore the value of guideline-based education as an effective approach to strengthen self-care practices essential for preventing avoidable foot complications. Overall, this study supports continued efforts to enhance patient education and training through the adoption of structured, nurse-led guideline interventions within routine care pathways. To improve generalizability, future research should be conducted with larger, probability-based samples drawn from multiple provinces in Saudi Arabia and other countries.

## Data Availability

The raw data supporting the conclusions of this article will be made available by the authors, without undue reservation.
